# Live Experiences of Adolescent Mothers Attending Mbale Regional Referral Hospital: A Phenomenological Study

**DOI:** 10.1155/2020/8897709

**Published:** 2020-11-20

**Authors:** Violet Chemutai, Julius Nteziyaremye, Gabriel Julius Wandabwa

**Affiliations:** ^1^Department of Obstetrics and Gynaecology, Busitema University Faculty of Health Sciences, Box 1460, Mbale, Uganda; ^2^Department of Community and Public Health, Faculty of Health Sciences Busitema University, Mbale Regional Referral and Teaching Hospital, Mbale, Uganda; ^3^Department of Obstetrics and Gynaecology, Faculty of Health Sciences, Busitema University, Mbale Town, Uganda

## Abstract

**Background:**

Adolescence is a period of transition from childhood to adulthood, and is a critical stage in ones' development. It is characterized by immense opportunities and risks. By 2016, 16% of the world's population was of adolescents, with 82% residing in developing countries. About 12 million births were in 15–19 year olds. Sub-Saharan Africa, particularly East Africa, has high adolescent pregnancy rates, as high as 35.8% in eastern Uganda. Maternal mortality ratio (MMR) attributable to 15–19 years olds is significant with 17.1% of Uganda's MMR 336/100.000 live births being in this age group. Whereas research is awash with contributing factors to such pregnancies, little is known about lived experiences during early motherhood. This study reports the lived experiences of adolescent mothers attending Mbale Hospital.

**Materials and Methods:**

A phenomenological study design was used in which adolescent mothers that were attending Young Child Clinic were identified from the register and simple random sampling was used to select participants. We called these mothers by way of phone numbers and asked them to come for focus group discussions that were limited to 9 mothers per group and lasting about 45 minutes–1 hour. Ethical approval was sought and informed written consent obtained from participants. At every focus group discussion, the data which had largely been taken in local languages was transcribed and translated verbatim into English.

**Results:**

The research revealed that adolescent mothers go through hard times especially with the changes of pregnancy and fear of unknown during intrapartum and immediate postpartum period and are largely treated negatively by family and other community members in addition to experiencing extreme hardships during parenting. However, these early mothers' stress is alleviated by the joy of seeing their own babies.

**Conclusion:**

Adolescent motherhood presents a high risk group and efforts to support them during antenatal care with special adolescent ANC clinics and continuous counseling together with their household should be emphasized to optimize outcome not only during pregnancy but also thereafter. Involving these mothers in technical courses to equip them with skills that can foster self-employment and providing support to enable them pursue further education should be explored.

## 1. Background

Adolescence is a period of transition from childhood to adulthood rather than an extended childhood period. It is described as a critical stage in one's development full of not only opportunities but also risks. It is therefore a period of natural experimentation, abstract thought, contemplating the future, empathy, and idealism yet characterized by immature decision-making [1]. World Health Organization (WHO) defines the age groups 10–19, 13–19, and 15–24 years of age as adolescents, teenagers, and youth, respectively. Those in the age group 10–24 years are called young people [1].

As of 2016, adolescents made up about 16% of the World's population with 86% of them living in the developing World. Approximately 21 million girls aged 15–19 years become pregnant in the developing world and about 12 million gave birth. At least 777,000 births occur to girls below 15 years of age in the developing world [2–4].

Adolescent pregnancy is very prevalent in Africa especially Sub-Saharan Africa. In a systematic review and meta-analysis of published and unpublished studies in Africa by Getachew et al., the overall prevalence of adolescent pregnancy in Africa was 18.8% and highest at 19.3% in Sub-Saharan African (SSA). East Africa registered the highest prevalence at 21.5%, while the North African region registered the lowest rate at 9.2% [5]. Moreover, a multilevel analysis of risk and protective factors of pregnancy and early motherhood among adolescents in five East African countries, in which the researchers focused on weighted subsample of adolescents aged 15–19  years pregnancy and early motherhood, was common in the five countries, ranging from 18% among adolescents in Kenya (2014) to 29% in Malawi (2016) and Zambia (2014) [6].

Adolescent pregnancy is associated with increased odds of maternal and perinatal adverse outcomes. Neonatal mortality increases as the age of the mother decreases; teenagers who give birth before the age of 15 years are five times more likely to die during pregnancy or delivery as women in their 20s, partly as a result of physical immaturity [7–9]. However, adolescent pregnancy and motherhood not only negatively affect the teenage mother in terms of health but also have far reaching physiological, psychosocial, economic, and cultural consequences such as dropping out of school, failure to reach ones potential, siblings likely to get pregnant, and ‘doorway' to poverty [7, 8, 10–13]. Some studies have held that it is a catastrophic period that literally prescribes one's ‘life script' [14].

The 2016 Uganda Demographic and Health Survey reported one of the highest maternal mortality ratios of the World at 336 per 100.000 live births and 17.1% of these were contributed by adolescents aged 15–19 years [15]. Although overall estimated prevalence of teenage/adolescent pregnancy in rural Uganda is at 27%, other regions especially Eastern Uganda continue to register rates higher than the national average, such as 35.8% in Kibuku [7, 15].

Factors associated with adolescent pregnancy including rural residence, ever married, being out of school, no maternal education, no father's education, lack of parent to adolescent communication on sexual and reproductive health (SRH) issues, divorce, contraceptive nonuse, multiple sexual partners, frequent sex, peer pressure, sexual abuse, and lack of control over sex were observed to increase the likelihood of teenage pregnancy [5, 16, 17].

Several studies have in fact urged that some adolescents have benefitted from early motherhood and become more responsive to positive living. It has further been urged that, once supported, these adolescent mothers can attain potential as much as their peers that postponed child bearing. Moreover debate has raged on that the undesirable lifestyles are not a consequence of adolescent pregnancies and motherhood but rather an extension of the predisposing factors [18–20].

However although studies have been done in different parts of the country to ascertain the factors associated with teenage pregnancies, there is a dearth of research concerning phenomenological studies to hear the adolescents' lived experiences.

## 2. Materials and Methods

### 2.1. Study Profile

80 potential female participants were selected and 65 purposively selected to take part in the study. The 65 were purposely selected in consideration of their residential areas being easily accessible to the Mbale Hospital. 20 were not able to turn up with the major reason being migration.
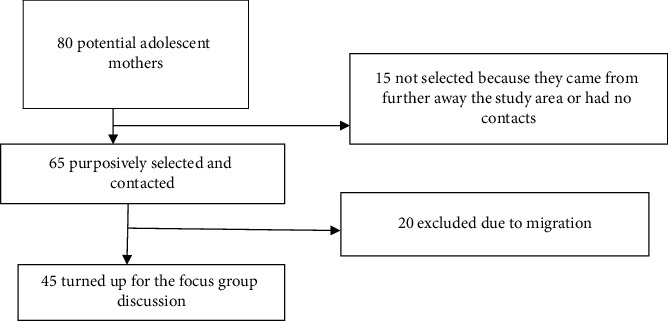


### 2.2. Study Design

We did phenomenological study design to collect qualitative data by way of focus group discussions (FGDS).

### 2.3. Study Area

The study was conducted from Mbale Regional Referral and Teaching hospital (MRRTH), Department of Obstetrics and Gynaecology Postnatal clinic. MRRTH is a 400-bed capacity hospital that serves about 14 districts in Eastern Uganda that include Bukwo, Bukedea, Budaka, Kibuku, Kumi, Kween, Kapchorwa, Sironko, Manafwa, Tororo, Namusindwa, Bududa, parts of Nakapririt, and Mbale. MRRTH offers specialized healthcare services and is a university teaching hospital for Busitema University Faculty of Health Sciences.

The Young Child Clinic on average attends to 30–50 mothers per day that come for child immunisation services, postnatal reviews, and family planning services. The hospital falls under Bugisu subregion that includes Bugisu region, including Mbale, Manafwa, Bududa, Bulambuli, Sironko, Kween, Bukwo, and Kapchorwa with teenage pregnancy prevalence of 28.2% [21].

### 2.4. Study Population

Adolescent Mothers who attended MRRTH Young Child Clinic

### 2.5. Selection Criteria

Using the clinic's register, we selected the participants by purposive sampling among those that had recently visited the facility with efforts taken to create diversity by inviting mothers from different parishes. The mothers were traced through their telephone contacts. If the availed number did not go through, the next coupon was picked till one was approached. The numbers used were either their own or ones of their relatives/friends as had been registered during the clinic day. We used the register since those mothers are at least 6 weeks postpartum and are thus more relaxed and likely to hold a discussion. The nature of the research was explained to the adolescent mothers and their voluntary participation was sought.

### 2.6. Sample Size

There was no sample size but FGDs were halted on realizing that saturation level had been reached.

### 2.7. Ethical Considerations

Ethical approval for this study was obtained from Cure Children's Uganda-Research and Ethics Committee (CCHU-REC/19/019) and administrative clearance sought from Mbale Regional Referral and Teaching Hospital (MRRTH). Informed consent was obtained from the research participants and participation was voluntary and efforts were taken to observe confidentiality. The participants were informed of voluntary nature of participation and ability to withdraw from the study at any time without undue explanations and that this would never jeopardize their ability to seek services from any department of MRRTH.

### 2.8. Data Collection and Management

Each focus group discussion was made up of 9 adolescent mothers and lasted for approximately one hour. In order to maximize openness and confidentiality, the research assistant used was not an employee in the clinic and had training in qualitative data collection. The research assistant had training prior to embarking on data collection. There were 10 guiding questions we used during the interview. These questions were developed for this research (supplement).

The data collection tool was pretested on other adolescent girls in the adolescent clinic. The focus group discussions were held in venues that were convenient for participants, such as open spaces at the health facility (after close of clinic hours). Lunch and transport refund were provided to the participants.

At every focus group discussion, the notes and recordings taken were largely in the local languages of Lumasaba, Lugwere, Luganda, and Ateso. Some participants communicated in English. After every focus group discussion, the recorded discussions were fully transcribed and translated verbatim into English [22]. Each transcript was analyzed by two researchers working independently to reduce bias using NVIVO software version 13. Coding was done manually based on the key words and phrases developed from the data. The codes were then grouped together into higher order headings. Accordingly, on a higher logical level of abstractions codes, subcategories, categories, and themes were formed. The themes were categorized according to the experiences in relation to pregnancy, child bearing, child care, parental views, and reactions. The data was sorted out thematically by clustering material with similar content. At this stage, we used a creative and analytical reasoning to determine categories of the meaning.

## 3. Results

Forty-five of the sixty-five adolescent mothers that were approached participated in the study.

20 were unable to come since they had relocated to places far away from Mbale.

The majority, 68.9%, of the participants had attained secondary level education and 31.1% had had primary level education. Moreover, the majority of the participants, 71.1%, were Christians, while the rest were Muslims. Furthermore, 71.1% were primiparous and only 28.9% were multiparous (Table 1).

### 3.1. Theme 1: Positive feelings

#### 3.1.1. Subtheme: Being a Young Mother Feels Good

About half of the participants expressed positivity about being a young mother.

“I feel good because am going to be called a mother,” said NA, 18 years old.

“I feel good because I have a child; who I will be sending to do something for me and respected as a mother in a community,” 19-year-old participant.

“It feels good because I have a child, there those who are looking for children but they have failed to get,” 17-year-old and 19-year-old participants.

“I feel good because I was not forced to get pregnant, it was my choice to have a pregnancy and I love to be called mummy,” 19 years old participant.

“I feel good because I love to be called mummy and the baby is mine,” 18-year-old participant

### 3.2. Theme 2: Negative Feelings

#### 3.2.1. Subtheme: Being a Young Mother Feels Shameful

In each FGD, some of the study participants reported that there was shame to have a baby at an early age and some expressed regret.

“I feel ashamed because people will laugh at me,” 18-year-old participant.

“I feel ashamed because my school mates will laugh at me,” 19-year-old participant.

“I feel ashamed in the community because of pregnancy though I have hope of getting the baby,” 19-year-old participant.

#### 3.2.2. Subtheme: It Feels Bad

In each of the discussion groups, participants mentioned they felt bad because people in the community went on talking about how they left school after conceiving their babies.

“I feel bad because people talk about me because I left school when still young” (19 years old).

“I feel bad because I left school and lost friends” (19 years old).

### 3.3. Theme 3: Fear of Reactions from Parents, Community, and Colleagues

The majority of the participants reported that their parents and guardians were not happy upon hearing the girl was pregnant; to many parents, pregnancies came as a surprise and they were upset and shocked. Participants had fear on how their parents would react upon hearing that they were pregnant.

#### 3.3.1. Subtheme: Reaction from Parents

*“I was mistreated by my parents, they did not want to be called grandparents because they are also still producing” (18 years old).*

*“I was treated like an outcast by both my parents and my community members because I had left fees at school” (17 years old).*

*“My parents did not want to be burdened with responsibility” (18 years old).*

*“My parents were happy after they realized I was pregnant because they knew dowry would be paid soon” (19 years old).*

#### 3.3.2. Subtheme: Community Reaction

Most of the study participants testified that the reaction from the community was very negative towards them including their friends.

“I was laughed at by my colleagues and felt so ashamed because the school fees had been misused” (19 years old).

“I have been denied respect from my community because I got pregnant at my age” (19 years old).

“Parents of my friends did not allow them to associate with me anymore because I am a spoilt girl. They feel their children may fall victims of early pregnancy” (18 years old).

“Some parents sent me away from their homes to avoid spoiling their daughters” (17 years old).

“They felt bad and sorry for her while other people were happy because she has also been brought down like others” (17 years old).

In one of the discussion groups, a participant reported that some of her community members felt so good upon her pregnancy because she had joined the group of drop-outs.

“Some of the community members felt good because I have joined them” (19 years old).

#### 3.3.3. Subtheme: Teachers' Reactions

Some of the participants reported that they felt bad and most especially the teachers.

“They felt very bad especially my teachers” (19 years old).

“They felt so sorry for me” (17 years old).

“The teachers and the community members started to talk against me when I got married” (18 year old).

“I and my teachers felt unhappy when I got pregnant at school” (19 years old).

“The teachers, parents and the community were not happy when I went to get married” (17 years and 19 years old).

“My teachers, guardians and friends were not happy and went police to report the case, my boyfriend was put in jail” (18 years old).

#### 3.3.4. Subtheme: In-Laws Reaction

Some of the participants mentioned that their in-laws and their parents were happy when they got pregnant. The in-laws expected them to take care of them, while some parents expected to receive dowry as it is a custom in the area.

“My in laws were happy that I was going to take care of them” (19 years old).

### 3.4. Theme 6: Reaction Based on Marital Status

Most participants reported that the unmarried teenage mothers are not given due respect by the community.

“They are not given any respect because they delivered while at their parent's home” (18 years old).

“They are being under looked, not loved and even friends have neglected them” (19 years old).

“They are being laughed at by the friends, that they will never marry” (17 years old).

“They are being abused and harassed because they expected a lot from them” (19 years old).

“They feel bad because they have dropped out of school” (19 years old).

All participants reported a negative feeling of being called a mother out of marriage as it was associated with shame and embarrassment to parents and the family at large.

“I fear my parents because they do not look at me with good attitude because I have had a baby while at home and Iam still young,” (18-year-old participant).

### 3.5. Theme 4: Teenager's Experiences during Labour and Immediate Postpartum Period

#### 3.5.1. Subtheme: Fear of Labour Pains

*“I had a lot of fear for labour pains and I have fear because I have no support for the baby,” 19-year-old participant.*

#### 3.5.2. Subtheme: Teenager in Labour

During the focus group discussions, the participants were asked to recall their experiences during labour and they all testified of pain they went through during labour.

“I felt a lot of pain that took 3 days before delivery of the baby” (19 years old).

“I felt a lot of pain but after delivery I was happier” (18 years old).

“I felt a lot of pain but I had hope of getting the baby” (19 years old).

“I felt bad during labour and regretted why I got pregnant. I swore never to get pregnant again” (18 years old).

“Because of the labour pains I regretted why I left school” (16 years old).

“I had fear and doubt whether I could deliver. Pain was too much” (18 years old).

“During labour I kept on calling onto GOD to help me… it was not my intention to get pregnant and I wish to be operated” (19 years old).

#### 3.5.3. Subtheme: First Sight of the Baby

Most participants testified that they had no regrets over carrying their pregnancy to term. Upon seeing their first time newborn, the pain and suffering vanished.

“I was happy to see my baby alive, I did not know I would make it” (17 years old).

“I have no regrets over having my baby, so glad and relieved” (19 years old).

“I am so happy to have a baby because there are people out yearning for children but they cannot have yet they have all the money” (18 years old).

“I am happy to have my baby girl, at least I have some joy in me because I was born alone and my parents are happy too” (16 years old).

### 3.6. Theme 5: Experience in Caring for the Baby

#### 3.6.1. Subtheme: Need for Family Support

Regarding the level of care the teen mothers gave to their babies, nearly all of them needed assistance from the family members during parenting. Many were supported by their mothers or older family members. Many admitted that they lacked parental skills and if it had not been the help offered by family members, they would not have coped.

“I was not able to hold the baby well during breastfeeding, I feared breastfeeding the baby, my mother supported me” (17 years old).

“My first day of breastfeeding I had no breast milk, the baby cried and I also cried” (19 years old).

“A baby cries, unable to speak, I do not understand why she is crying, I tried to make my baby happy and show love to the baby” (19 years old).

“I was filled with joy when I saw my baby breast feeding for the first time” (17 years old).

“I feel good and happy when my child is not sick, breastfeeding well” (19 years old).

“I had hard time in starting to take care of the baby” (19 years old).

A few of the participants mentioned that they had no support from their partners; they showed no love, no attention, and no support towards the caring of the child.

“I wanted to eat after delivery but I had nothing” (18 years old).

“I lack a source of income for supporting the baby, medication, feeding and clothing” (19 years old).

“I have no assistance from the man, he is very rude to me” (16 years old).

#### 3.6.2. Subtheme: Stress

Two of the participants report that there was stress in caring for the baby.

“I feel a lot of stress in caring for the baby like who to feed, clothing the child and medication of the baby” (19-year-old participant).

“It's hard and stressing to have a child at this age because I was not prepared for the child” (19-year-old participant).

### 3.7. Theme 7: Abortion

#### 3.7.1. Subtheme: Option of Abortion

One of the participants mentioned that she had opted to carry out an abortion although she failed to procure one. She narrated that this would have been a better option taken to dodge community embarrassment. Most young girls do abortion without knowledge of their parents or guardians.

“As teenage girl I had fear and opted to abortion the baby to avoid being away from home to the boy's home” (19-year-old participant).

### 3.8. Theme 8: Effects of Teenage Pregnancy

The participants in each of the discussion groups mentioned that pregnancy affects them not only physically but also psychologically, physiologically, and emotionally.

#### 3.8.1. Subtheme: Physiologically

*“Teenage pregnancy leads to damage of the reproductive system because they are still young and developing” (17 years old).*

*“It affects them especially during labour” (19 years old).*

*“Some of the teen mothers are getting problems by attempting abortions, they get infected with diseases like HIV/AIDS, syphilis and even lose life because of excessive bleeding” (19 years old).*

*“It gave me complications of the uterus, I failed to deliver normally, I was taken to theatre to remove the baby” (16 years old).*

#### 3.8.2. Subtheme: Emotionally

*“Lack of enough support affects them emotionally and psychologically” (19 years old).*

*“I have passed through hard life, he denied me and I have no support now from him” (19 years old).*

#### 3.8.3. Subtheme: Education

*“It affected my education, I did not complete even primary level” (18 years old).*

*“My future plans and goal has been affected” (17 years old).*

#### 3.8.4. Subtheme: Psychologically

*“There is no good planning because aim still young” (16 years old).*

*“I developed ulcers at a young age because my husband is not caring for me and the child” (19 years old).*

### 3.9. Theme 9: Reasons for Getting Pregnant

#### 3.9.1. Subtheme: Financial Support

Lack of support for school fees was one of the reasons for getting pregnant.

The study findings revealed that some participants got pregnant in search of support from the boyfriends who had money because the parents were unable to support them financially and materially.

“It was because my parents were unable to pay her school fees, I got someone (man friend) who supported in paying my tuition and bought my scholastic materials and eventually I became pregnant,” echoed 17 and 19 years olds.

“I had no much help from my parents so I felt my boyfriend will do much for me” (19 years old).

“It's because of the situation, my parents were not giving me a enough support, the solution was to get someone to support me” (19 years old).

#### 3.9.2. Subtheme: Peer Pressure

A few of the teenage mothers mentioned that they got pregnant due to peer pressure. The friends could advise them to try it to show love to the boyfriend.

“I got pregnant due to peer pressure from the peer groups” (19 years old).

“I had pressure from peer groups of opposite sex, who told me let's just try and I got pregnant” (19 years old).

“I had wrong peer groups who led me into trouble” (19 years old).

#### 3.9.3. Subtheme: Orphans

In the two focus group discussions, some participants mentioned that they are orphans, parents died when they were still young and these rendered them vulnerable to teenage pregnancy. The guardians and grandparents were unable to support them while at school.

“I am an orphan, my parents passed on when I was still young, my guardians took care of me but they could not support me at school financially so I ended up in the hands of a man who tried to support but made me pregnant before I completed school” (19 years old).

“I am an orphan, my grandparents could not support me at school and I was left with no choice but to get married” (19 years old).

#### 3.9.4. Subtheme: Parental Pressure

In one of the group discussions, one of the participants mentioned that she had parental pressure. The parents wanted dowry and since the girl respected her parents, she had to go for marriage.

“My parents wanted dowry and I had no choice” (19 years old).

#### 3.9.5. Subtheme: Sexual Experimentation

Some of them confessed having been stubborn; they could not heed to the parental advice and went on their own engaging and experimenting in sex. They preferred to explore and receive advice from the peers than the parents. Adolescence is a rebellious stage where the teens are so wild; do not listen to counseling even when parents are able to provide everything they needed. Their stubbornness led them into pregnancy.

“I failed to listen to parents' advice, I became too stubborn. My boy friend used to give money and these worsen everything and I thought I had everything. I could not turn down my boyfriend's proposal till he made me pregnant” (19 years old and 18 years old).

“I felt it was time to try these things and we had competition due to high demand of good things which the boy friend could provide” (19 years old).

We also explored the ways they thought adolescent pregnancy could be prevented in the community, what services an adolescent mother could expect to receive while at the health facility, and ways government can help the affected mother.

### 3.10. Theme 10: Ways They Thought Adolescent Pregnancy Could Be Prevented

Most participants from each discussion group mentioned that teenage pregnancy could be prevented through health education, abstinence, family planning, being patient, counseling and guidance, listening to parental advice, avoidance of wrong peer groups, education of girl child, and provision of basic needs to girls by the parents.

“Teen pregnancy can be prevented through health education about the dangers of early pregnancies” (18 years old).

“Teenage pregnancy could be prevented by abstaining from sex until the right age reaches” (19 years old).

“Use of family planning methods and patience could help in prevention of teenage pregnancy in our community” (15 years and 19 years old).

“Teenage pregnancy can be prevented through counseling the teenage girls by telling them reality of what happens to them. Tel them of the real problems she has gone through so that they do not fall victims of circumstances” (17 years old).

“Girls should avoid bad peer groups and listen to advice from parents” (16 and 18 years old).

“Parents should provide young girls with basic needs” (15 years old).

### 3.11. Theme 11: Suggestions on Services Teenage Mothers Expect to Receive from the Health Facility

A number of study participants suggested that they wished to receive Maama kits, parental love and care, counseling and guidance, strengthening of free treatment services, health education and counseling on care of the baby, family planning, mosquito nets, and enough number of staffs to attend to them. Mama Kit is a basic kit for normal delivery that is distributed to all expectant mothers attending antenatal care clinic in public facilities in Uganda but is also sold. It contains essential items for a clean and a safe delivery by mothers [23].

“We expect to be given mama kits during our pregnancy from this health facility” (19 years old).

“We expect to be given parental love and care from the health facility by both the doctors and the midwives and not shouting at them” (19 years old).

“I expect to receive counseling and guidance by the health workers from the health facility” (17 years old).

“I expect to receive proper examination by the nurses/midwives and the doctors for all the patients” (18 years old).

“I expect to receive health education and counseling on how to care for the baby and nutrition” (19 years old).

“I expect the health facility to be giving family planning advice to the young girls” (15 years old).

“Mothers should be given mosquito nets, free treatment” (17 years old).

### 3.12. Theme 12: Suggestions on How the Government Should Help Those Already Affected

#### 3.12.1. Pregnant or Already Have Children

From all the focus group discussions, almost all participants gave suggestions on how government should help the already pregnant teens or having children in order to improve their standard of living.

“Government should provide enough essentials like mama kits and drugs,” said a 17-year-old participant.

“Government should offer for opening up projects that could help them generate some income for sustainability in the community,” echoed a 19 years old.

“The government should treat us equally with the rest of people in the country,” said a 17 years old.

“We request the government to support those who want to go back to school as teen mothers in terms of school fees and scholastic materials,” said a 19 years old.

“The government should get sponsors to support them in skills development/training,” a 19 years old retaliated.

“The government should strengthen giving free medical services for all,” said a 16 years old.

“The government should strengthen the law against defilement in the country,” said an 18-year-old participant.

“The government should provide basic education for a girl child,” said a 15-year-old participant.

## 4. Discussion

Being an adolescent/teenage mother brings with it different feelings and challenges. What is directly known is that these adolescents have got enormous responsibilities in addition to their needs as adolescents [19, 24]. Research has shown that these challenges not only affect the current generation but also spill over to the next generations [25].

Although it has been a long time held view that adolescent pregnancy is catastrophic in ones' life [14], research also suggests that it may actually form the ‘backbone' and turning point of one's life from that describable as ‘irresponsible' to a more responsible and meaningful life, once one is offered the necessary support [18, 26].

In our study, several adolescents expressed positive feelings of becoming a mother, being looked at as one who has grown up in the community and having achieved what other adults had strived to achieve but in vain. Being a mother felt good. This finding was similar to one in a study in Australia among the African born refugee [27], Belgium [28], and one in Ghana [29]. Motherhood, however, also brought some mixed feelings and experiences. It was associated with stress, fear of labour pains, damage to the reproductive system, and anxiety of caring for the baby. This was further compounded by lack of the partners' support in addition to disapproval by community members, family, and rejoicing teenagers that had earlier dropped out of school for various reasons. Research elsewhere has shown similar findings and reported that spouse support, immediate family support, and school administrators support were critical in elevating these young ladies' self-esteem, resumption of school, and completion of college education [18, 24, 27].

In our study, the findings indicated that the community negatively perceived adolescent pregnancy and such a girl was described as spoilt and thus not worth interacting with, especially by the nonpregnant adolescents, not worth wasted school fees, an outcast by parents and community, and a source of shame to their families. This was similar to findings in studies in Ghana and South Africa [29, 30]. These findings are echoed by the adolescent girls themselves who stated that the community was more positive of one who conceived after marriage, at age above 18 years (legal age for marriage in Uganda) [31]. In this study, the adolescents further described what the culturally acceptable sequence of events is: marriage and then pregnancy and therefore deviation from this norm was typically met with a negative view of the adolescent girl and her pregnancy [13, 31]. The negative sentiments by the community are not only in Africa but also have been expressed elsewhere, though still in the same population of Africans [27].

This finding further agrees with a study in South Africa that shows that generally African cultures disapprove of adolescent pregnancies [32]. The negative view is further amplified by the adolescents' expression of repercussions of adolescent pregnancy on school attendance as expressed in other studies. Moreover, related research has shown that keeping girl child in school is a way of averting adolescent pregnancy and motherhood [20, 33, 34]. The stigma starts form the girl herself to the school community. Some may be expelled or asked to return after giving births [14, 31]. A study by Kaye further stressed that even when these adolescents come to health facilities, they are stigmatized by health workers [11]. This finding is in contrast to the one by Phoua Xiong among the Hmong communities because the latter takes it as a norm [25]. But it is worth noting that the acceptance in the Hmong community is for ‘face saving' in order to create harmony [35].

The disapproval of teenage pregnancy by the community and friends leads to these mothers contemplating and at times executing abortions [29, 31, 32]. These negative sentiments coupled with restrictive laws on procurement of abortion services are sought to undermine efforts aimed at curtailing maternal mortality [36–38]. In our study like in the above studies, some girls had attempted to abort but failed.

Poverty was highlighted as one of the reasons these girls got pregnant. They expressed their parents' desire for dowry, failure of the parents to provide tuition, scholastic materials, and other basic needs. This affirms that poverty is a driver of adolescent pregnancy and childhood marriages [29, 39]. This finding further emphasizes the concept of transactional sex phenomenon in Uganda and other parts of Africa [29, 31].

### 4.1. Reasons for Becoming Pregnant

A score of participants explained that it is their experimentation of sexual intercourse that got them into pregnancy. They confessed to not listening to parents' advice but rather relying on their peers. Their peers advised them to have sexual intercourse as a sign of love. In addition to sex experimentation and peer pressure, others blamed this on orphanage and parent pressure. These latter two were all related to the inability of the parent or guardian to provide for the adolescent girl, while in some cases the parents wanted dowry. These findings were similar to a study in Ghana where adolescents expressed the same reasons, but, in contrast to Ghana study, there was no adolescent who got pregnant because she did not know that indulging in sex could get one pregnant [29].

Despite the physiological and economical vulnerability of these adolescents, they managed to carry their pregnancies till delivery. Some though expressed regret dropping out of school, being labeled as outcasts, spoilt and money wasters, and lacked support from partners and community. These sentiments are in agreements with studies elsewhere [19, 29, 31].

Their economical vulnerabilities were further expressed in the lack of utilities when they visited public health facilities. Several of these adolescent mothers suggested that economic empowerment projects, especially those that encompass technical skills, and social and moral support aimed to help the mother return to school after giving birth, and strengthening the defilement law would better the girl child. Worth noting is that Uganda has got a very stringent law on defilement [40] but with a lot of implementation challenges especially due to weak legislation and out-of-court settlements between the plaintiff and offender [41, 42].

## 5. Conclusion

The study builds our understanding of the unique features, challenges, and desires of these adolescent mothers, which services to provide to them, and ways of how to address the problem from their own perspective. It further emphasizes the need for champions in parent/s' love and provision of counseling and family planning services in the fight against adolescent pregnancy. Most are willing to go back to school and pursue their goals and thus need tremendous family and community support. Adolescent mothers further thought that strenthening and implementaion of the defilement laws would offer more protection against the vice. The study further showed that most of these adolescents are in consensual relationships and rarely attempted abortion.

## 6. Suggested Areas of Research

What are the lived experiences of adolescent fathers?

## 7. Strength of the Study

We demonstrated that adolescents have a say in their pregnancy and could help us design the programs that benefit them during antenatal care and in everyday life thereafter especially equipping them with skills.

## Figures and Tables

**Table 1:** Characteristics of participants.

Variable	Number (%)
Age	
15 yrs	3 (6.7)
16 yrs	6 (15.4)
17 yrs	5 (11.1)
18 yrs	11 (24.4)
19 yrs	20 (44.4)
Education	
Primary	14 (31.1)
Secondary	31 (68.9)
Occupation	
Employed	3 (6.7)
Unemployed	42 (93.3)
Religion	
Christian	32 (71.1)
Moslem	13 (28.9)
Parity	
Primiparous	32 (71.1)
Multiparous	10 (28.9)
Who she lives with	
Living with husband/boyfriend/cohabiting	36 (80)
Not living with husband/boyfriend/cohabiting	9 (20)

## Data Availability

The data used to support the findings of this study are available from the corresponding author upon request.

## Abbreviations

ANC: Antenatal care
MRRTH: Mbale Regional Referral and Teaching Hospital
SSA: Sub-Saharan Africa
SRH: Sexual and reproductive health
UDHS: Uganda Demographic and Health Survey
WHO: World Health Organization.
